# Possibility of Using Flexible Dentures over Iliac Bone Graft in Adolescent Patients with Ameloblastoma: A 9-Month Follow-Up Clinical Report

**DOI:** 10.1155/2021/2415707

**Published:** 2021-11-18

**Authors:** Ammar Belal, Bassil Monther, Wael Alzarif

**Affiliations:** ^1^Master's Degree, Clinical Instructor, School of Dentistry, Hama University, Removable prosthodontic Department, Hama, Syria; ^2^Professor, Doctorate, School of Dentistry, Hama University, Removable prosthodontic Department, Hama, Syria; ^3^Oral and Maxillofacial Surgery, Syrian Board of Medical Specialties, Head of Oral and Maxillofacial Surgery, National Hospital of Hama, Hama, Syria

## Abstract

**Introduction:**

The use of bone grafts is a common procedure after excision and reconstruction of the mandible, although it is rare in children and adolescents due to incomplete growth, which means a long transition period until reaching an appropriate age for implants or more predictable outcomes. *Case Report*. This article describes a 9-month follow-up of the use of a flexible denture above a bone graft taken from the anterior iliac crest for adolescent patients with resected mandible due to ameloblastoma. Taking into account prosthetic considerations, radiography, and clinical observation, no complications were seen with the graft.

**Conclusion:**

It is safe to use a flexible denture as a prosthetic over an iliac bone graft block during the healing period.

## 1. Introduction

Ameloblastoma is a rare tumor, but it affects all ages, especially between the second and fifth decades, and equally, in both sexes, its incidence in the lower jaw is 80% compared to the upper jaw. Although it is a benign tumor, it has a pandemic effect because it grows without symptoms and tends to be recurrent in 90% of conservative approach (enucleation, marsupialization, or curettage) [1–4]. Therefore, to get the best results, 1 to 2 cm of healthy bone around the tumor must be excised, whatever its type (multicystic, peripheral, and unicystic tumors) [5–7]. This excision may lead to a dramatic collapse of the patient's swallowing, pronunciation, chewing, and aesthetic functions, and the loss of anatomical structures will make the procedures of prosthetics difficult, especially in cases of the lower jaw, in addition to the deviation of the remaining segment toward the defect side which may lead to the loss of occlusal contacts on the defect side and a shift in the posterior functional contacts on the normal side [8, 9]. Therefore, expeditiously rehabilitation after marginal or segmental mandibulectomy is preferred [10, 11]. There are many techniques for the management of mandibular continuity defects such as nonvascularized bone graft, iliac crest free flap, and vascularized osseous free graft [12]. Whatever the donor site is (the fibula, scapula, rib, and the iliac crest) [13], it is desirable to preserve the bone graft until full healing to make the final prosthetic whatever its types including implant-supported fixed prostheses [14], or removable dentures whether supported by implants or not [15, 16], but until then, the bone graft must be protected in addition to helping the patient during this stage to restore the masticatory, aesthetic, and verbal function, as well as improving his psychological state through the interim prosthesis, which is mostly a removable denture. Although interim care is not indicated for mandibular defects, most cases and discussions usually talk about maxillary ones; unless, they have to wait for the surgical site to heal and if it is dimensionally stable [17, 18]. Many studies describe the factors influencing the success or failure of bone grafts especially those related to surgery or postsurgery procedures such as type of defect and scope of excision, bone graft infection, extrusion, malocclusion, facial nerve involvement, or deformation of lower face [19, 20], while factors related to the prosthetics are studied extensively in terms of its effect on alveolar bone resorption in conventional cases, such as its type, design, method of impressions, or occlusal scheme [21–25]. Prosthodontics considerations in cases of mandibular resection were mentioned in articles and references that also mentioned about acrylic and hybrid prosthetics due to their ability to be modified with an interim or resin lining material; they also talked about their design such as not extending the base widely as normal dentures, reducing the occlusal surface, and the use of existing teeth for additional retention in metal or acrylic dentures [18, 26–28]; however, it is rare to find an article about using the flexible dentures maybe because the controversy about it is not over yet; anyway, it is still a viable option (polyamide, polyester, acrylic resin, polycarbonate, and polypropylene); in addition to having many useful advantages and indications, interim dentures or spare dentures for patients with metal allergy or for whom esthetics must be given top priority [29]. The purpose of this clinical report is to describe the possibility of using interim flexible acrylic dentures over iliac bone graft for a considerable period in cases of mandibular resection in adolescent patients with ameloblastoma.

## 2. Clinical Report

**Figure 1:**
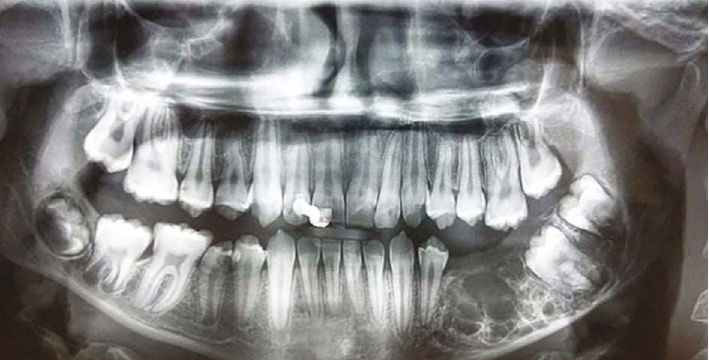
Preoperative panoramic radiograph.

**Figure 2:**
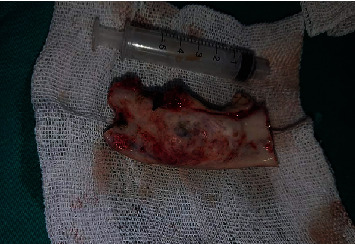
Excised bone.

A 12-year-old male patient reported to the Department of Oral and Maxillofacial Surgery at the National Hospital of Hama with visible swelling; intraorally, there was a noticeable expansion of the mandible body in the posterior section. After the radiographic examination, the presence of multicystic ameloblastoma was suspected (Figure 1), which was confirmed by histological examination. On the CBCT scan, the tumor was extending from tooth 34 to tooth 38 with part of the ramus. According to Ord et al. [30], the treatment of ameloblastoma would be complicated because of continued growth and a higher percentage of cancellous bone which increased bone turnover and high periosteal reactivity, so the treatment should be in the same way used with adults; therefore, a partial resection of the mandible from the tooth 33 until the ramus was done while keeping the condyle (Figure 2).

**Figure 3:**
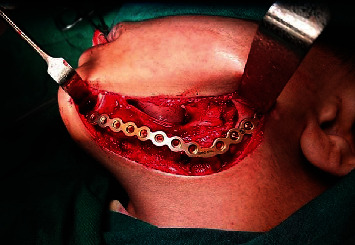
The titanium reconstruction plate.

**Figure 4:**
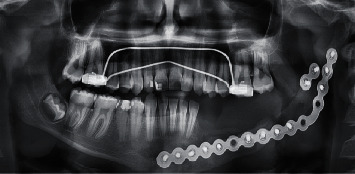
Postoperative view with the graft.

A titanium reconstruction plate was adapted and contoured to the mandible body to reinforce and help stabilize a block graft, but we waited 8 months before applying the bone graft to ensure that there was no recurrence of excision, and due to the lack of technology required to operate a microsurgical reconstruction beside, the patient was unable to travel to another place where these experiences are available, so a nonvascularized bone graft was taken from the anterior iliac crest, and because the patient's bone is spongy and fragile, we waited a whole year, and after that, we confirm the success of the graft and the stability of its dimensions. During this period, a transpalatal arch was applied to prevent rotation of upper molars and maintain the arch width (Figures 3 and 4). The second stage of treatment involved a removable partial denture until the patient reaches an appropriate age to place implants and fixed prosthetic where the additional bone graft may be applied before placing the implants.

**Figure 5:**
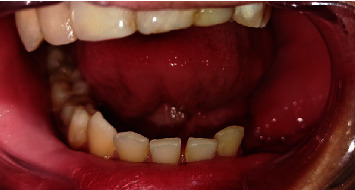
Intraoral view.

Intraoral examination showed an obvious volume of soft tissue in the iliac graft region which had healed well (Figure 5), and all options were discussed with the patient's parents regarding their economic situation or expectations who refused to perform another surgical procedure to remove the flabby soft tissue, so the prosthodontic plan was designed to fabricate a flexible partial denture.

**Figure 6:**
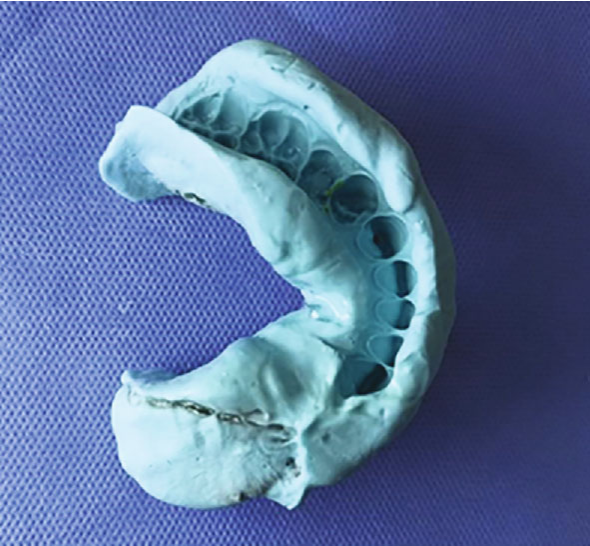
Final impression.

As usual, the procedures began with making a primary impression using irreversible hydrocolloid material. The custom tray was made by autopolymerizing acrylic resin (Simplex Hi, Kemdent, UK) and checked in the patient mouth. To fill the missed area of the resected mandible in the tray, impression compound sticks (Kerr, Italy) were used to support the final impression material and to make the border molding; then, the functional impression was made with irreversible hydrocolloid material (Zetaplus, Zhermak, Italy) and poured with the pink gypsum type IV (Shera, Italy) (Figure 6).

The occlusal relationship was registered using a register plate made with autopolymerizing acrylic resin and modeling wax (Tenatex, Kemdent, UK) to make the rim that holds the medium (Chemi Sil Bite, Hyvincare, Korea), taking into account the situation of the mandible and its deviation, and because this stage is considered critical, the relationship was manually directed until reaching the most stable position, taking advantage of the remaining teeth on the natural side, and then was confirmed during the clinical trial.

**Figure 7:**
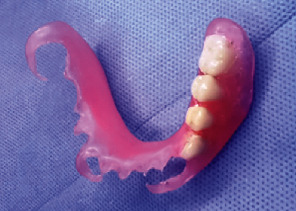
The flexible denture.

**Figure 8:**
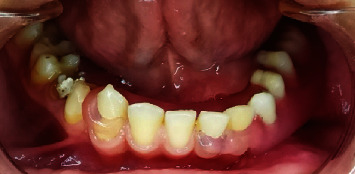
Flexible dentures inside the mouth.

The denture was processed with flexible acrylic resin (Valplast, Tianjin Iris, China) and delivered to the patient after adjustment procedures and assessment of both speech and mastication (Figures 7 and 8); after that, the artificial teeth were prepared according to class I of black and restored with an amalgam filling to create a natural look for a young man of this age.

**Figure 9:**
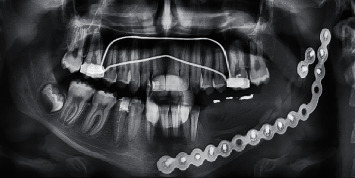
A radiographic image after denture delivery.

A digital panoramic radiograph was made with a silicone bite on the biting block of the radiographic device to ensure that the patient bites the same situation after the end of the observation period which was 9 months (Figure 9).

## 3. Discussion

The initial plan chosen for this patient was a flexible partial denture that will be used for a considerable period. All options for the type of the removable partial denture have been taken into account, starting with the cast metal one. But it is not considered a logical option due to the incomplete growth of young patient besides its high price, especially with the possibility of making a new denture after a while, in addition to the difficulty of controlling the lever effect around the rest with the presence of flabby tissue which may cause damage to the bone graft or natural teeth, as leaving the patient without a denture in this age is also illogical because of the need to restore the mastication and cosmesis in addition to the possibility of food trapping and possible infections of the surgical area due to the difficulty cleaning or impaired neuromuscular function [17]. The second option was a heat polymerizing resin denture as an interim prosthetic due to its cheap price and lining capacity, but the presence of the large volume of flabby tissue and the difficulty of obtaining sufficient stability or the possibility of damage to adjacent teeth and the health of supporting soft tissues [17], especially above the graft area, make the flexible denture a more acceptable choice for several reasons:

- Its flexibility allows obtaining additional stability and retention by increasing the flange extension under hard or soft tissue undercuts, in addition to extending the clasp of more teeth [24, 29]
- Softer surface compared with acrylic resin means that patients feel better when wearing it and there appears to be no problem with the fit [ 24, 29]
- Thermoplastic resins have hygienic advantages due to their low water absorption and solubility [24]
- Aesthetically, it is better than acrylic resin [24]

Taking into account some of its disadvantages, such as difficulty to repair or poor resistance to scratching [24], the patient was alerted to the need for oral hygiene and regular reviews.

**Figure 10:**
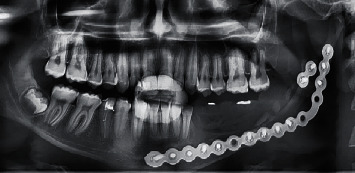
After 9 months.

To reduce the functional efforts transferred to the graft by reducing the total occlusal load applied in the supporting tissue, the lingualized articulation at the premolars was adopted but anatomic one at the first molar. For more effective chewing but with the deletion of the second molar [17, 24], panoramic imaging is an accepted procedure for observing vertical changes in the bone [31], which after nine months showed no bone resorption or has not exceeded bone remodeling (Figure 10); besides, the intraoral examination showed healthy tissue with no wasting or any infection.

## 4. Conclusions

Within the limits of this case and the duration of observation of a bone graft taken from the anterior iliac crest under a flexible partial denture, adhering to prosthetic considerations, it can be said that it is safe to use a flexible denture as a prosthetic over iliac bone graft block during the healing period [32].
